# Challenges and opportunities: CAR-T cell therapy in autoimmune diseases

**DOI:** 10.1016/j.cellin.2026.100327

**Published:** 2026-04-24

**Authors:** Ran Yan, Songying Ye, Huji Xu

**Affiliations:** Department of Rheumatology and Immunology, National Key Laboratory for Immunity and Inflammation, Shanghai Changzheng Hospital, Naval Medical University, Shanghai, 200003, China

**Keywords:** CAR-T cell therapy, Autoimmune diseases, Allogeneic CAR-T cells

## Abstract

Chimeric antigen receptor T (CAR-T) cell therapy is rapidly moving from hematologic malignancies into severe autoimmune disease, particularly B cell-mediated disorders such as systemic lupus erythematosus (SLE), systemic sclerosis (SSc) and inflammatory myopathies. Recent autologous and allogeneic studies show that deep immune-cell depletion can induce drug-free remission or major clinical responses in the refractory patients. However, translating CAR-T cell therapy into autoimmune disease raises challenges that differ from oncology, including target selection in heterogeneous immune ecosystems, depletion of nonpathogenic immune compartments, control of cytokine release syndrome and infection risk, definition of the optimal degree of CAR persistence, and scalable manufacturing. In this review, we summarize the major obstacles and emerging opportunities for CAR-T cell therapy in autoimmune diseases, with emphasis on representative clinical studies, candidate targets beyond CD19, engineering strategies to improve safety and durability, and the specific promise and limitations of allogeneic platforms.

Chimeric antigen receptor T (CAR-T) cell therapy has emerged as a revolutionary treatment modality in the field of immunotherapy. Initially developed to treat cancers, particularly hematologic malignancies, it has now shown promising clinical effect in the treatment of autoimmune diseases such as systemic lupus erythematosus (SLE), systemic sclerosis (SSc) and myositis. However, the adoption of CAR-T cell therapy in autoimmune diseases is not without its challenges. The technology's application in autoimmune diseases requires a tailored approach, focusing on antigen specificity, safety, persistence and controllability of engineered cells. This article reviews the major challenges associated with CAR-T cell therapy in autoimmune disease and discusses where the field may be heading next.

Recent clinical work has moved the field beyond proof of concept. The initial five-patient autologous anti-CD19 CAR-T series in refractory SLE established that deep B-cell depletion can induce drug-free remission (Mackensen et al., 2022), the phase 1/2 CASTLE basket trial extended this experience to SLE, SSc and idiopathic inflammatory myopathies (IIM) (Müller et al., 2026), and several allogeneic T-cell and NK-cell platforms have shown promising activity in SLE, SSc and immune-mediated necrotizing myopathy (Wang et al., 2024, 2025a, 2025b, 2025c). In parallel, dual-targeting strategies such as co-infused CD19/BCMA CAR-T cells and BCMA-CD19 armored compound CAR-T cells are being explored to address plasma-cell-mediated persistence (Feng et al., 2025; Hong et al., 2026), while antigen-specific chimeric autoantibody receptor (CAAR) T cell approaches such as MuSK-CAART illustrate how the field may eventually move from broad B-cell depletion to precision depletion of pathogenic clones (Oh et al., 2023).

Representative clinical studies discussed in this review are summarized in Table 1. To keep the table focused, we included representative clinical studies that have already entered autoimmune-disease application; preclinical or translational studies such as MuSK-CAART are discussed in the main text but are not listed in the table.

**Table 1:** Representative clinical studies of CAR-based cell therapy in autoimmune diseases.

Study	Platform	Disease	Key efficacy signals	Key safety/translational points
Mackensen et al. (2022)	Autologous anti-CD19 CAR-T	Refractory SLE (*n* = 5)	All 5 reached DORIS remission at month 3; drug-free remission persisted despite B-cell return after 110 ± 32 days	Only mild CRS
Müller et al. (2026)	Autologous anti-CD19 CAR-T	SLE (10), SSc (9), IIM (5)	22/24 met efficacy endpoints; SLE 9/10 DORIS remission; SSc 9/9 no progression; IIM 4/5 ACR major/moderate response	No CRS > grade 2; no ICANS (immune effector cell-associated neurotoxicity syndrome); 7 patients required intravenous immunoglobulin substitution
Wang et al. (2024)	Allogeneic anti-CD19 CAR-T	IMNM (1), dcSSc (2)	Complete B-cell depletion within 2 weeks; deep remission during 6-month follow-up	No CRS or other serious adverse events
Wang et al. (2025b)	Hypoimmune allogeneic CD19 STAR-T (YTS109)	SLE with LN (*n* = 5)	All 5 achieved SRI-4 (SLE responder index-4) response at month 3, sustained through month 6; renal biopsies showed resolution of inflammation and tissue restoration	Mild CRS only; no GVHD
Wang et al. (2025a)	Allogeneic anti-CD19 CAR-T	Severe refractory SLE (*n* = 3)	SELENA-SLEDAI and SLEDAI-2K declined; all 3 reached SRI-4 at the last visit	No GVHD, CRS or ICANS; no severe adverse events recorded
Feng et al. (2025)	Co-infused autologous anti-CD19 + anti-BCMA CAR-T	Treatment-refractory SLE (*n* = 15)	By week 12, 12/15 fulfilled both LLDAS and DORIS remission; 1-year monitoring in a subset suggested sustained eradication of pathogenic clones	Grade 1 CRS in 86.7%; no neurotoxicity or treatment-related deaths
Hong et al. (2026)	BCMA-CD19 armored compound CAR-T (ICG318)	Refractory SLE (*n* = 12; 10 with LN)	10/12 achieved stringent complete remission; 11/12 achieved drug-free serological remission; durable responses approached 6 years	No high-grade CRS or neurotoxicity
Wang et al. (2025c)	iPSC-derived CD19/BCMA CAR-NK (QN-139b)	Severe dcSSc (*n* = 1)	Reversed fibrosis and restored skin and vascular structure; reduced autoantibodies and depleted pathogenic B cells	Minimal toxicity

## 1 Challenges in CAR-T cell therapy for autoimmune diseases

### 1.1 Antigen target selection in autoimmune diseases

One of the significant challenges in CAR-T cell therapy for autoimmune diseases is the selection of suitable target antigens. Unlike cancer, where specific tumor-associated antigens (TAAs) are more easily identifiable, autoimmune diseases are driven by a range of self-antigens that target normal, healthy tissues. These antigens may vary significantly among patients and across different autoimmune conditions.

Identifying a single or a few specific antigens that are exclusively present on the pathogenic cells of autoimmune diseases, without affecting healthy tissues, is a formidable challenge. For instance, in SLE, B cells are typically the primary target, but the disease manifests with numerous autoantibodies against a wide range of self-antigens. This means that CAR-T cells must be engineered to target specific immune cells, such as autoreactive B cells or T cells, while avoiding off-target effects on essential, healthy cells.

Furthermore, the heterogeneity of autoimmune diseases adds another layer of complexity. In diseases such as SSc or myasthenia gravis, the pathogenic cells and antigens involved are not as easily identified as in B-cell malignancies. Thus, there is a pressing need for ongoing research to identify more precise antigen targets in the context of these diseases (Buckner, 2025; Collet et al., 2025).

Current target development follows at least three tracks. First, CD19 remains the most clinically validated target because it covers most stages of the B-cell lineage and can induce a broad immune reset (Buckner, 2025; Mackensen et al., 2022; Müller et al., 2026). Second, BCMA-directed or CD19/BCMA dual-targeting strategies attempt to extend depletion to plasmablasts and long-lived plasma cells, which may be especially important in autoantibody-driven diseases such as SLE and lupus nephritis (Feng et al., 2025; Hong et al., 2026). Third, antigen-specific CAAR approaches, exemplified by MuSK-CAART for MuSK myasthenia gravis, aim to eliminate autoreactive B cells while preserving the remainder of the B-cell compartment (Oh et al., 2023). The unresolved question is which diseases require broad depletion, which require layered targeting of both B cells and plasma cells, and which are mature enough for precision autoantigen-specific therapy (Buckner, 2025).

### 1.2 Off-target effects and toxicity

Off-target effects represent a major safety concern in CAR-T cell therapy. In autoimmune diseases, the immune system's attack on self-tissues leads to chronic inflammation and tissue damage. When CAR-T cells are deployed, there is a risk of their recognizing and attacking healthy cells that express low levels of the targeted antigen, even if these cells are not the primary drivers of the autoimmune response. This phenomenon could lead to unwanted destruction of healthy tissues, exacerbating the patient's condition or causing additional harm.

For example, in CAR-T cell therapy for autoimmune diseases such as systemic lupus erythematosus, there is a possibility that CAR-T cells may target B cells that are not pathogenic, or that have a critical role in maintaining immune homeostasis. This could result in immune suppression, leaving the patient vulnerable to infections and other complications. Furthermore, the destruction of normal cells due to off-target effects can lead to life-threatening conditions, including organ failure.

The risk of cytokine release syndrome (CRS), a common side effect in CAR-T cell therapy, is particularly prominent in autoimmune diseases. CRS occurs when CAR-T cells are activated and release a massive influx of inflammatory cytokines, leading to symptoms such as fever, hypotension, and neurological complications (Brudno and Kochenderfer, 2024). Although anti-IL-6R antibodies like tocilizumab have been used to mitigate CRS, its occurrence remains a significant concern, especially in patients with autoimmune diseases, where immune regulation is already compromised (Berry et al., 2025; Yu et al., 2024).

Importantly, the problem of collateral depletion is no longer only theoretical. In CASTLE, serum IgG, IgA and IgM decreased after CAR-T cell therapy, and seven patients (29%) required intravenous immunoglobulin substitution because of higher-grade hypogammaglobulinemia or recurrent mild infections. At the same time, most vaccine-induced IgG titers remained stable, suggesting that profound B-cell depletion does not necessarily erase all protective humoral memory. These observations strengthen the argument for more precise targeting and for prospective monitoring of immunoglobulins, vaccine serologies and infection burden in future trials (Müller et al., 2026).

### 1.3 Persistence and longevity of CAR-T cells

The persistence and longevity of CAR-T cells are vital for the sustained effectiveness of the therapy. In cancer therapies, the goal is often to completely eradicate tumor cells, but in autoimmune diseases, long-term immune modulation is more desirable. However, CAR-T cells often suffer from issues related to their persistence and functionality after infusion. The immune system may recognize the engineered T cells as foreign, leading to their early destruction.

Clinical data indicate that optimal persistence is context dependent rather than simply maximal. In the initial five-patient SLE series, all patients achieved DORIS remission at 3 months and maintained drug-free remission despite B-cell return after a mean of 110 ± 32 days, supporting a transient 'deplete-and-reset' model in at least a subset of patients (Mackensen et al., 2022). In CASTLE, CAR-T cells were no longer detected after a median of 83 (56-113) days, yet most patients maintained clinical responses throughout the 24-week primary observation period and remained free of glucocorticoids and other immunosuppressive treatment over that period (Müller et al., 2026). These data support the concept that durable remission can persist after relatively limited CAR detectability in at least some patients (Mackensen et al., 2022; Müller et al., 2026).

However, transient depletion may not be universally sufficient. Liu et al. recently argued that, in genetically determined SLE, recurrent disease-driving biology may re-establish autoreactive B-cell programs after CAR-T disappearance. This work suggests that some monogenic or continuously antigen-driven disease states may require sustained CAR activity, repeat dosing or other maintenance strategies rather than a one-time reset approach (Liu et al., 2026). For autoimmune medicine, this implies that persistence goals should eventually be individualized according to disease biology, tissue reservoir burden and tempo of immune reconstitution.

Concrete strategies to enhance persistence are also emerging. Preclinical work shows that engineered CAR-enhancer modules can increase CAR-T-cell proliferation, memory formation and re-expansion upon rechallenge, illustrating one route to improve durability when longer surveillance is needed (Rakhshandehroo et al., 2025). Although these strategies were developed mainly in oncology, they provide actionable design principles for autoimmune CAR-T programs that seek sustained immune control without indiscriminate long-term immunosuppression (Labanieh and Mackall, 2023).

### 1.4 Manufacturing and cost considerations

The process of manufacturing CAR-T cells is both time-consuming and expensive, posing a significant barrier to the widespread use of this therapy. For autologous CAR-T cell therapy, the patient's own T cells must be extracted, genetically modified, and expanded before being re-infused. This process can take several weeks, during which time the patient may experience disease progression.

Moreover, the production of CAR-T cells is highly individualized, requiring custom manufacturing for each patient. This leads to logistical challenges, as well as concerns about manufacturing failures and delays. The high cost of CAR-T cell therapy is another limiting factor, with treatment costs reaching hundreds of thousands of dollars per patient.

These pressures have stimulated strong interest in allogeneic and other off-the-shelf platforms. However, faster access and lower batch-to-batch cost are only one part of the solution; the field must also solve allo-recognition, persistence, genomic editing quality control and scalable release testing if such products are to become broadly deployable (Li et al., 2025; Li and Yang, 2026; Wu et al., 2025).

### 1.5 Regulatory and approval challenges

The regulatory landscape for CAR-T cell therapy in autoimmune diseases is still evolving. While CAR-T cell therapy has received approval for certain hematologic malignancies, its use in autoimmune diseases is still in early stages. The lack of established clinical guidelines and regulatory frameworks for autoimmune indications poses a barrier to its widespread adoption.

Future regulatory development in autoimmune CAR-based therapy will likely require robust evidence not only for short-term efficacy but also for durability, immune reconstitution, infection risk and late toxicities. These requirements are likely to shape trial design, patient selection and pharmacovigilance strategies as the field moves from first-in-human studies toward broader clinical adoption (Li and Yang, 2026; Scherlinger et al., 2025; Wu et al., 2025).

## 2 Allogeneic CAR-T cells: Unique challenges and opportunities

Allogeneic CAR-T cell therapy, which uses genetically modified immune cells from healthy donors instead of the patient, offers an especially attractive strategy for autoimmune diseases because it could shorten manufacturing time, reduce logistical burden and improve access (Li and Yang, 2026). This opportunity has rapidly shifted from theoretical to clinical. Healthy-donor-derived CD19 CAR-T cells achieved deep remission in one patient with refractory immune-mediated necrotizing myopathy and two patients with diffuse cutaneous SSc without CRS or other serious adverse events over 6 months (Wang et al., 2024). In refractory SLE complicated by lupus nephritis, YTS109 induced SRI-4 (SLE responder index-4) responses in all five treated patients at month 3, sustained to month 6, with only mild CRS and no graft-versus-host disease (Wang et al., 2025b). Another allogeneic CD19 CAR-T study reported durable remission with 12-month follow-up in three patients with severe SLE and no GVHD, CRS or ICANS (immune effector cell-associated neurotoxicity syndrome) (Wang et al., 2025a). In addition, an iPSC-derived CD19/BCMA CAR-NK product produced marked B-cell depletion and reversal of fibrosis with minimal toxicity in a patient with severe SSc (Wang et al., 2025c).

### 2.1 Graft-versus-host disease (GVHD)

One of the major risks associated with allogeneic CAR-T cell therapy is the development of graft-versus-host disease (GVHD), a condition in which the infused donor T cells attack the patient's tissues. This occurs when the donor T cells recognize the recipient's tissues as foreign and mount an immune response against them. GVHD can lead to severe complications, including tissue damage, organ failure, and death.

Researchers are therefore using multiplex gene editing and locus-controlled engineering to reduce alloreactivity (Engel et al., 2025; Jiang et al., 2025). In recent autoimmune disease studies, editing strategies have included disruption of endogenous T-cell receptor signaling and modification of antigen-presentation pathways to reduce both GVHD and host-versus-graft recognition (Wang et al., 2024, 2025b). The success of these platforms suggests that allogeneic feasibility depends on engineered immune compatibility rather than donor substitution alone (Wang et al., 2024, 2025b).

### 2.2 Immune rejection of allogeneic CAR-T cells

Another issue is the potential for immune rejection of allogeneic CAR-T cells. Since these cells are derived from a donor, they may be recognized as foreign by the patient's immune system, which could lead to their rapid destruction. This immune clearance can reduce the persistence and efficacy of the CAR-T cells, limiting their therapeutic potential.

To address this issue, researchers are developing strategies to "mask" the allogeneic CAR-T cells, making them less recognizable by the recipient's immune system. One approach involves the use of gene editing to disrupt human leukocyte antigen (HLA) expression on CAR-T cells, which could reduce immune recognition and improve cell persistence (Jiang et al., 2025; Labanieh and Mackall, 2023).

### 2.3 Manufacturing and scalability

While allogeneic CAR-T cells offer an "off-the-shelf" solution, their production is still complex and costly. The need to ensure that the donor cells are healthy and free from infections or other complications remains a significant hurdle. Additionally, large-scale manufacturing processes must be established to meet the demand for these cells, particularly as the treatment becomes more widely adopted.

Scaling up the production of allogeneic CAR-T cells involves overcoming challenges related to cell expansion, quality control, and regulatory approval. Moreover, the high cost of producing and manufacturing these cells remains a barrier to their widespread use, particularly in resource-limited settings (Li and Yang, 2026; Wu et al., 2025).

## 3 Future prospects and research directions

### 3.1 Target antigen optimization

The future of CAR-T cell therapy for autoimmune diseases lies in more rational target optimization. Current evidence suggests that at least three target-design logics will coexist. One is broad B-cell depletion, exemplified by CD19 (Mackensen et al., 2022; Müller et al., 2026). A second is layered targeting of B cells and plasma cells, exemplified by CD19/BCMA combinations or armored compound designs (Feng et al., 2025; Hong et al., 2026). A third is precision autoantigen-specific targeting, exemplified by CAAR strategies (Oh et al., 2023). Which logic is preferable will likely depend on whether the dominant disease engine resides in circulating B cells, long-lived plasma cells or narrowly defined autoreactive clones (Buckner, 2025).

### 3.2 Enhancing safety profiles

Improving the safety profile of CAR-T cell therapy is essential for autoimmune indications. In addition to limiting CRS and off-target depletion, future products should ideally include built-in mechanisms that allow rapid control or elimination of the engineered cells if severe toxicity occurs.

One major direction is the development of synthetic control modules that can gate or terminate CAR-T-cell activity in time and space. Recent reviews of synthetic-switch platforms summarize kill switches, adaptor switches, drug-responsive circuits and other spatiotemporal control systems that are being developed to reduce CRS, neurotoxicity and on-target/off-tumor injury (Lu et al., 2024).

Another promising approach is the development of armored CAR-T cells. In cancer, the term often refers to CAR-T cells engineered to secrete cytokines or resist inhibitory microenvironments. In autoimmune disease, one clinically relevant armored design is broader coverage of the humoral disease axis. The BCMA-CD19 armored compound CAR-T product ICG318, for example, was designed to target both CD19-positive B cells and BCMA-positive plasma cells or long-lived plasma cells, and extended follow-up suggests that this broader targeting can support highly durable medication-free remission in refractory SLE (Hong et al., 2026).

### 3.3 Allogeneic CAR-T cells and “off-the-shelf” solutions

The development of allogeneic CAR-T cells presents an exciting opportunity to reduce the cost and improve the accessibility of CAR-T cell therapy for autoimmune diseases. With allogeneic CAR-T cells, the need for individualized manufacturing is eliminated, and treatment can be initiated more quickly. However, as previously mentioned, allogeneic CAR-T cells come with their own set of challenges, including the risk of GVHD and potential immune rejection.

The next step will be to refine these platforms for autoimmune indications specifically. This includes optimizing editing architecture, balancing immune invisibility against persistence, and determining whether T cells, NK cells or iPSC-derived effectors are best suited for particular disease contexts (Wang et al., 2024, 2025b, 2025c).

### 3.4 Long-term persistence and immune modulation

Enhancing persistence and immune-modulating properties will be key to achieving long-term remission in autoimmune diseases, but, as discussed above, persistence should be tuned to disease biology rather than maximized indiscriminately.

Future CAR-T therapies may therefore need to combine mechanism-based patient stratification with platform-level persistence engineering (Buckner, 2025). In practice, this could mean selecting shorter-lived immune-reset strategies for some patients, while reserving more durable platforms or repeat-dosing concepts for genetically determined or continuously antigen-driven disease (Liu et al., 2026; Müller et al., 2026). Preclinical CAR-enhancer data and emerging autoimmune clinical observations together support this more individualized view of persistence (Liu et al., 2026; Rakhshandehroo et al., 2025).

### 3.5 Broadening the scope of CAR-T cell therapy

As research continues to evolve, CAR-T cell therapy may expand to a broader range of autoimmune diseases, including both systemic and organ-specific antibody-mediated disorders. The pace of this expansion will depend on how clearly pathogenic immune-cell populations can be defined and how acceptable the risk-benefit ratio is in diseases that already have multiple approved therapies (Scherlinger et al., 2025; Wu et al., 2025).

### 3.6 Future perspectives and development trends

In our view, three trends are likely to shape the next phase of the field. First, indication selection will shift from diagnosis-based enrollment toward mechanism-based patient stratification for cell therapy, with the strongest early rationale in diseases that are clearly B-cell or plasma-cell driven and clinically refractory (Buckner, 2025). Second, target design will move from single-antigen pan-B-cell depletion toward layered precision, including dual B-cell/plasma-cell targeting and, where feasible, antigen-specific CAAR approaches (Feng et al., 2025; Hong et al., 2026; Oh et al., 2023). Third, platform development will increasingly prioritize manufacturability and controllability, bringing together multiplex immune editing, hypoimmune allogeneic products, iPSC-derived effectors and embedded safety switches (Engel et al., 2025; Li and Yang, 2026; Lu et al., 2024). Future trials will need to pair clinical endpoints with deep immune monitoring so that clinicians can distinguish patients who need transient immune reset from those who may need sustained cellular surveillance (Liu et al., 2026; Scherlinger et al., 2025).

## 4 Conclusion

CAR-T cell therapy has moved from a provocative concept to an early clinical reality in autoimmune disease. Representative studies now show that both autologous and allogeneic platforms can induce deep immune reset and clinically meaningful remission in selected patients with severe refractory disease. At the same time, the field still faces major questions regarding target specificity, collateral immune depletion, persistence requirements, manufacturing scale-up and long-term safety. The most important near-term goal is not simply to reproduce oncology CAR-T paradigms in autoimmune disease, but to define which patients, targets and platform designs are biologically appropriate for different mechanistic contexts of autoimmune disease. If these challenges are addressed, CAR-based cell therapy may become a transformative option for otherwise untreatable autoimmune disease.

## CRediT authorship contribution statement

**Ran Yan:** Writing – original draft, Investigation, Data curation. **Songying Ye:** Writing – original draft, Investigation, Data curation. **Huji Xu:** Writing – review & editing, Supervision, Resources, Conceptualization.

## Declaration of competing interest

The authors declare that they have no known competing financial interests or personal relationships that could have appeared to influence the work reported in this paper.
